# Shadow Puppets and Neglected Diseases (2): A Qualitative Evaluation of a Health Promotion Performance in Rural Indonesia

**DOI:** 10.3390/ijerph15122829

**Published:** 2018-12-12

**Authors:** Courtney Williams, Donald E. Stewart, Dan Bendrups, Budi Laksono, Joko Susilo, Salvador Amaral, Johanna Kurscheid, Darren J. Gray

**Affiliations:** 1 Queensland Conservatorium Research Centre, Griffith University, South Brisbane, QLD 4101, Australia; cewilliams13@gmail.com; 2 School of Medicine, Griffith Health, Griffith University, South Brisbane, QLD 4101, Australia; 3 Graduate Research School, La Trobe University, Victoria 3083, Australia; D.Bendrups@latrobe.edu.au; 4 Yayasan Wahana Bakti Sejatera Foundation (YWBS), Semarang 50183, Indonesia; dokterbudilaksono@gmail.com; 5 Music Department, Theatre and Performing Arts, Otago University, Dunedin 9016, New Zealand; dhalang@yahoo.com; 6 Department of Global Health, Research School of Population Health, Australian National University, Canberra, ACT 2600, Australia; salvadorcoro@yahoo.com (S.A.); Johanna.Kurscheid@anu.edu.au (J.K.); Darren.gray@anu.edu.au (D.J.G.)

**Keywords:** soil-transmitted helminths, health promotion, shadow puppetry, knowledge and behaviours, Indonesia

## Abstract

Performing arts used as a method of spreading health information dates back to the origins of storytelling. However, interventions in developing, non-Western countries typically utilize Western entertainment forms. This qualitative investigation assesses responses to an intervention designed around traditional Javanese shadow puppetry (*wayang kulit*). Semi-structured interviews provided in-depth responses from a sample (N = 12) of villagers. Responses analyzed both cross-case and within-case, focused on perceptions of the music and storyline, responses to the intervention, and the perceived appropriateness of *wayang kulit* for disseminating a health message. *Wayang kulit* was considered to be interesting and easy to remember, but concerns remained regarding the reliability of information provided through the drama. The fusion of traditional and modern music and story elements were perceived positively. Some participants were inspired to improve their hygiene practices, although the lack of motivation, or belief that they were unable to change was noted. The performance was generally received positively in terms of the nature of the intervention, the fusion of traditional and Western music and story elements, as well as the use of *wayang kulit* to spread health information. The study provides guidance for modifications to the production, prior to scaling up.

## 1. Introduction

The concept of integrating education and the performing arts has roots dating back to the origins of storytelling and examples of this practice are found across the world [1]. Over the last two decades, the concept of ‘Entertainment-Education’ (E-E) or ‘Edutainment’ has evolved into “the process of purposely designing and implementing a media message to both entertain and educate in order to increase audience members’ knowledge about an educational issue, create favorable attitudes, shift social norms, and change overt behavior” [2]. Two primary theories underlie research into the effectiveness of E-E interventions [3]. The social cognitive theory suggests that people learn vicariously through models and these models should be both attractive and relatable to the audience and have their positive behavior reinforced and negative behavior corrected. The extended elaboration model postulates an alternative—that it is the audiences’ engagement with the dramatic narrative that reduces their critical state and likelihood to counter-argue, priming them to be more receptive to new beliefs, attitudes, and behavior. In terms of enhancing the effectiveness of E-E interventions both models are useful. For instance, the emphasis on the importance of positive role models by Chesterton [4] can be seen to fit within the framework of social cognitive theory. On the other hand, other recommendations such as those of Piotrow and de Fossard [5] support the extended elaboration model, arguing, for example, for a careful balance between entertainment and education and the importance of credible plots, characters, and dialogue. Likewise, Singhal and Rogers [2] argue that the effectiveness of E-E is related to emotions, which are the base of behavior-change triggers. Thus, for instance, the grief over character sickness and seeing how this ill-health affects others is more likely to trigger behavioral change than rationally-structured media messages.

E-E interventions have targeted hygiene in developing countries. In Nepal, for example, a government initiative sent out one-way SMS messages encouraging handwashing [6,7]. The children’s animated series *Keremet Koch* tackled health and social issues, with post-test results showing that hygiene was one of the most frequently recalled issues by viewers [8]. The Meena Communication Initiative, a multifaceted approach including animated films, posters, radio series, and discussion/teacher guides, all of which were designed to address social and health issues (including hygiene and sanitation, worm infestation and prevention), was based on comprehensive formative research, including focus groups and revision by technical experts. In Nepal, this initiative saw positive behavioral change in 96% of children who were aware of Meena, the most popular of which were hand washing and encouraging siblings to maintain personal hygiene [4].

E-E interventions have specifically targeted neglected tropical diseases such as the reduction of parasitic diseases. For instance, the 12 minute educational animated video intervention the “Magic Glasses” focused on preventing soil-transmitted helminth (STH) infections in schoolchildren in Asia [9]. First developed for and evaluated (via a cluster-RCT) in Hunan Province, China, the “Magic Glasses” intervention was highly successful, with a 50% decrease in the incidence of STH infection (OR = 0.5, 95% CI 0.35–0.7, *p* < 0.0001) in the intervention schools compared with the control schools [10]. These results were unprecedented for educational interventions targeting STH, and provided proof of principle that the video-based health educational package widens student knowledge and changes behavior, resulting in fewer STH infections. The “Magic Glasses” has now been adapted to the Philippines and Vietnam and is currently undergoing evaluation in these settings.

While such interventions have been effective, there is still scope for further development. Despite the acknowledged importance of accounting for culture and tradition in E-E interventions [5], such interventions most commonly utilize Western entertainment forms, especially radio and mass-mediated television formats [2]. The intervention at the center of this research project was specifically designed to engage with Javanese audiences by incorporating traditional media forms culturally familiar to village audiences. However, an innovative aspect was that it also employed a soundtrack with elements drawn from Western, commercial music production, thus straddling both (external) Western and (local) Javanese domains of cultural production.

## 2. Methods

### 2.1. Study Context

This study is part of a larger investigation into improving health and sanitation practices in Central Java [11]. A companion article that focuses on the quantitative analysis of a larger sample of village participants has been published by this journal [12], investigating environmental conditions, as well as knowledge, attitudes, and behaviors regarding parasites and STH in particular. Using a pre/post design, statistically significant improvements were found at follow-up in both knowledge and behavior. In the current paper, we take a very different perspective and focus on cultural and ethno-musicological issues, examining whether the meaning of the intervention conflicted with or was compatible with *wayang kulit* as an art form. Our research investigates the reflections of a selected group of participants on whether they were comfortable about the use of the epic ‘Ramayana’ narrative as a medium for health education; about their reaction to the fusion of traditional gamelan and Western ‘jazz’ musical instruments; about the story-line and narrative climax; and about whether the shadow puppet medium was considered as appropriate and authentic for health education purposes.

Previous research by Stewart and Laksono [13] identified several reasons for the lack of success surrounding latrine campaigns, including the impact of wet and dry seasons on the availability of water for human waste disposal, the need for cultural familiarity, ease of use, and affordability. This is in line with research by McLeroy, Bibeau, Steckler, and Glanz [14], which argues that health interventions need to look beyond targeting the individual, and towards the environment and society as a whole, as individuals have limited capacity to control their total health environment. An intervention was then developed, introducing squat latrines (the ‘BALatrine’), alongside a health education/health promotion component focusing on hygiene and sanitation. The latter component included village-wide, small group, and one-on-one meetings, including discussion and the distribution of pamphlets covering how the BALatrine can improve health and how to construct, use, and maintain the BALatrine [15]. The initial phase of this project was successful, with the introduction of the BALatrine linked to reduced levels of water contamination, soil-transmitted helminths (STH) infection, and bowel-disease related absence from work or school [16,17].

**Figure 1:**
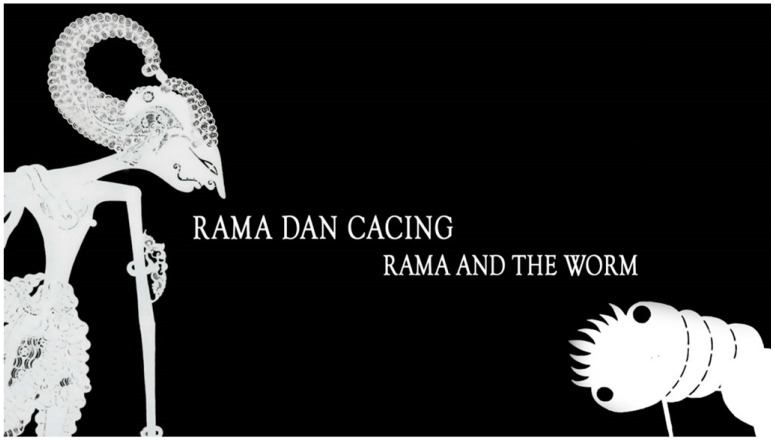
Title credit for ‘*Rama and the Worm*’ shadow puppet production.

Despite this, it was evident that further work was required to promote the systematic use of the BALatrine [18]. Accordingly, we expanded the health education component to use traditional Javanese shadow puppet theatre, *wayang kulit*, as a resource to disseminate health messages surrounding hygiene and use of the BALatrine. With one of our investigators (JS), a fifth-generation *dhalang* (puppet master) from Solo, Central Java, we had a unique opportunity to create a production that was culturally sensitive and appropriate for our intended audience. The resulting new work (see Video S1): *‘Rama and the Worm’* (*RATW*), available in a low-res English language version on YouTube at https://youtu.be/AafNmmMlrvQ, or Bahasa Indonesia version at https://youtu.be/0-TEOOaTG0A, was designed to combine both traditional Javanese and Western elements in a way that was both distinctive and attractive to a Javanese audience [18]. The title credit for the production is shown in Figure 1 below. 

This qualitative study presents an appraisal of *RATW* by a cross section of villagers in central Java, Indonesia, analyzing data obtained from in-depth interviews with males and females across the age range. Semi-structured interviews designed to obtain a clear, contextualized understanding of the reception of *RATW*, focused on the clarity of the messages, the reception of the traditional and modern fusion of the music and story elements, and the effectiveness of the shadow puppet medium to convey health messages. In order to ensure that participants were not providing ‘expected’ responses to our interviewers, and recognizing that in many communities polite conversation is one that is agreeable to ‘visitors’ and may not necessarily reflect true feelings or emotions, we conducted rigorous training sessions with cross-checking follow-up or subordinate queries.

### 2.2. Study Design

In line with current qualitative methodology, we anticipated that the first six interviews would uncover new ideas or thoughts relating to RATW and that ‘saturation’ would be reached prior to the 12th interview [19]. This was indeed the case, as became apparent when preparing the analysis. Due to the context of the study and the need to assess the effectiveness of *RATW*, we chose template analysis as a primary method of analysis [20]. Here, predetermined codes are created from the questions asked during the interviews. Due to the open nature of the questions, we used open coding to identify sub-categories within these codes. After each transcript was read, codes were added and redefined until it was thorough enough to build an account of the findings. When creating codes, themes were informed by health promotion outcomes recommended by Nutbeam [21], especially as relating to improved health literacy: knowledge, attitudes, motivation, behavioural intentions, personal skills, and self-efficacy.

Consistent with findings by Guest, Bunce, and Johnson [19], new themes did not appear after the first half dozen interviews and, due to the targeted nature of the questions, saturation was reached well before the analysis of the final interview. In order to further enhance the validity through methodological triangulation, responses were analyzed using both a cross-case, thematic analysis, and then within-case using Systematic Text Condensation (STC) [22]. The STC analysis allowed for a more narrative, contextual perspective within the analysis.

The principal objective of this research was to consider the reception and effectiveness of this novel approach to health promotion, seeking views on the content, storyline, music, and dramatic quality of the production. Thus, we took a *gestalt* approach and the analysis of the reception and responses to *RATW* were framed within a cultural context. Additionally, the analysis acknowledged the presence of the researchers, as recommended by Boeije [23], by incorporating prompting/leading questions into the analysis. This added layer to the contextual approach allowed insight into whether answers were a neutral representation of the participants’ true beliefs, or if they were designed to please the researcher—an issue that can arise in interview-based qualitative research [20].

### 2.3. Participants, Materials and Procedure

The project was discussed and approved by critical decision-makers in the community, including local leaders (village heads) and education specialists. The format of *wayang kulit*, a traditional Javanese theatre practice involving the use of shadow puppets, was chosen as the medium through which to relay the health messages. It was made available to audiences in the form of a short (c. 20 minute) film. Featuring stories and characters drawn from Indian epic tales that are well known in Java, *wayang kulit* was chosen as it has also been used as a medium to incorporate contemporary issues and events, with non-traditional puppets being introduced into shows.

Susilo and Bendrups set out to develop a new narrative that wove the message into a narrative developed from extant *wayang* storylines based on the *Ramayana*. Integrating the health message into a traditional plot this way allows the audience to become familiar with and identify with these characters and thus be more likely to implement changes within their own lives [5]. The following story was created, based upon the *Ramayana* framework:

*Ravana, a demon devotee of the warrior god Shiva, is angered by the noise and laughter made by an industrious town. His assistant suggests enlisting the help of parasites* [here, the concept of Soil Transmitted Helminth (STH) was introduced]*, and so Ravana casts a spell allowing him to shrink, and bargain with the parasites. Prince Rama, an avatar of the god Vishnu who had been exiled into the forest, came across this village, which had been laid waste by STH. A villager pleads with Rama for help, and so Rama investigates, and discovers the source of the problem.* [The causes for the increase in parasites—i.e., village growth, open defecation, poor personal hygiene—is taught by Rama here]*. Rama shrinks, goes inside one of the children to confirm the diagnosis, and finds Ravana inside the boy’s stomach. Rama and Ravana fight, and losing, Ravana hides with the worms. Rama battles a giant STH* [more information on STH is provided here]*, eventually defeating several worms and Ravana. Rama leaves the boy’s stomach, returns to normal size, and explains what has happened to the villagers. Rama then gives a call to arms, instructing the villagers in how to protect themselves from worms in the future by following his three main messages.*

Musically, *RATW* incorporated the traditional *wayang* accompaniment of the gamelan ensemble. Modern rock and pop instruments (bass guitar, brass, keyboard, etc.), were gradually incorporated into the score, until they dominated during the climactic fight scene, in order to increase the sense of drama and urgency.

*RATW* was introduced and screened at a general community meeting in a sub-village, or ‘*dusun*’, where all villagers were invited to attend. Twelve interviews were then conducted, with an equal distribution of males and females and a selection across the broad age range of villagers: 4 younger villagers (5–25 years); 4 adults (26–45 years); and 4 older adults (46 years and over). The sub-village community (Candi Mulyo) selected is located in Wonosobo, in the Province of Central Java—an area that has been identified as one in which a substantial proportion of village households lack ‘improved latrines’, following the WHO definition as being a facility that hygienically separates human excreta from human contact. One dataset was excluded from analysis, as it was evident that the participant had given answers that were heavily influenced by the interviewer’s questions. As initial analyses revealed that saturation had already been reached by this point, it was deemed unnecessary to find a replacement participant.

Semi-structured interviews were conducted using an interview checklist (see Supplementary Materials S2), and were conducted by interviewers from the local area in a friendly village atmosphere. These interviewers underwent a comprehensive training course, and gave the participants the option to conduct the interview in either Bahasa Indonesia, or the local language, Javanese.

The study received ethics approval from the Griffith University Human Research Ethics Committee (2016/442) on 26 June, 2017. Written informed consent was obtained from all participants, data were de-identified and anonymous and all participants were given the opportunity to withdraw without penalty, should they so wish.

## 3. Results

### 3.1. Effectiveness and Appropriateness of ‘RAMA and the Worm’

A major objective of the study was to investigate if *RATW* had any relationship with (or relevance to) village practices. Two of the participants stated that it directly related, while four believed that certain aspects did relate to what went on in their village. The participants’ views were changeable, however, with four participants changing their response after personal reflection, or after being prompted by the interviewer to elaborate on their answers.

Where participants stated that the story did not relate to their village, this was primarily related to their belief that open defecation was not much of a problem in their community. They stated that they (or others) defecated in an appropriate place (n = 5), and/or that their village had public lavatories (n = 3). Aspects of *RATW* that were considered relevant were that that people do not wash hands (n = 3), that children play in the river (n = 2), and that people litter (n = 2). Overall, it was difficult to gain a clear picture of village practices, as many justifications associated with the relationship (or lack thereof) between the issues portrayed in *RATW* and the participants’ own village contradicted each other (for instance, whether or not lavatories were used, and whether or not children played/bathed in the river).

These responses are understandable in terms of the village context where household disposal of human waste is primarily through use of a non-flushing latrine (usually a hole in the floor) over a fish pond that flows under the house and out to open street drains or culverts. ‘Public’ latrines in this context are small shelters over the street drain/culvert in front of the house, where passing pedestrians can relieve themselves. The definitional difficulties regarding what is an ‘improved latrine’ (see above) and what is regarded as a latrine by village dwellers has already been addressed by the authors in some detail [11].

In addition to investigating the perceived relationship between *RATW* and village practices, the interview questions also investigated participants’ beliefs as to the appropriateness of *wayang kulit* medium for disseminating health messages. Initially, it appeared that most (n = 6) of the participants’ comments showed they agreed that the *wayang kulit* was well suited to this function, however, two participants (upon being prompted) later changed their response. Where *wayang kulit* was considered appropriate, this was primarily related to cultural involvement or preservation of culture, and the preference of *wayang* as a traditional medium, especially “for older people—because *wayang* is older people’s favourite” (female participant, age 16).

Participants that found *wayang kulit* appropriate reported that this was because it was easier to understand and easier to concentrate upon than non-entertainment-based methods of health education. As one commented: “Because I can more listening and more concentrating by video, because I can watch while listening. So those two things can be remember through the running image and the voice can quickly understand” (male participant, age 21). This suggests that participants were engaged in the narrative, in line with the theory that E-E is underpinned by the extended elaboration model [3]. This was not the case for all participants, with a number (three) not comfortable with the innovative fusion—not purely traditional Javanese, and two recognizing that it was not entirely ‘traditional’.

A separate issue identified by six of the participants was that they would have preferred the narration for *RATW* to be in local Javanese, not Bahasa Indonesia. The decision to provide narration in Bahasa was taken in order to make this pilot version of the film as widely accessible as possible, but the project team took this decision with a suspicion that the use of the national, official language may be undesirable to some viewers at the local level. The participants’ response confirms this and lends weight to an argument for interventions such as *RATW* to incorporate additional support for local language versions.

Another factor arising from the discussion of the appropriateness of *RATW* was that some participants described the film as being appropriate for others, but not for themselves. One participant, for instance, commented that they believed they were able to judge when they were at risk, stating that “after eating, if I feel my hand still clean, yaa, I do not wash my hand, anyway I ate using spoon. My hands still clean after eating, so I would not wash my hand” (male participant, age 21). Similarly, one participant stated that the story related to the village in that—as in the story—the children of their village play in the river, but unlike the story, they contended that “the river is clean, here, not dirty” (female participant, age 34). Another insisted that worms were not the cause of stomach aches and diarrhoea in their village, but “that must be symptoms of spiciness” (male participant, age 49). This cognitive bias towards underestimating their own risk (or ‘perceived invulnerability’) is a challenge that is acknowledged in extant literature [3]. In light of this feedback, a future iteration of *RATW* might benefit from the further foregrounding of a relatable, vulnerable character, such as the ‘villager’ who appears briefly in the third scene.

A further issue that arose was that, as a medium for disseminating the health message, *RATW* was perceived by some participants as ‘less convincing’ than other forms of health education. For some, it was unclear whether *RATW* was portraying real events, or merely an entertaining story:

It’s not sure that the story on the video is not correlated with our daily activities *mba* [nickname for older woman]. Not sure that all people will understand the video, because the story of *wayang* is like not sure, so, people just watch it and so … they do not know if the story is for real.

(Male participant, age 43)

This view was reinforced by the participants’ further statements about their preferred medium for receiving health education. Only three of the participants preferred *wayang kulit* for this purpose, with the rest preferring a direct explanation, doctor/allied health services (n = 6) or a pamphlet/book (n = 2). Doctors and allied health professionals were preferred for their perceived clarity, accuracy, knowledgeability, modernity, trustworthiness, and convincingness, while books and pamphlets could be saved and re-read. One participant, for instance, described how “the health services are more convincing, because they are used to explore in the health issue, so they must be more knowledgeable and more convincing” (male participant, age 21). This lack of trust in the information provided in the entertainment is an acknowledged challenge of E-E interventions [3]. However, practical solutions to this issue were proposed by several of the participants, who suggested that the film could be made more effective by combining it with companion presentations by health or allied health professionals, pamphlets to be distributed at future screenings, or the inclusion of a “health message present in the beginning before video played” (female participant, age 15).

### 3.2. Participants’ Habits and Intentions to Change

The main focus of the research was to obtain rich detail regarding audience responses to the use of a traditional medium for health messages about sanitation and hygiene and prevention of infection by parasites, however, additional data were collected about whether the participants intended to change their habits after watching *RATW*. There was a mixed response to this ‘intention to change’ issue. Six of the eleven participants affirmed that they did, while five stated that they did not intend to change (although three of these participants later contradicted their answers). One participant provided some insight into the reasons inhibiting their intention to change, affirming that they did not believe that they were able to change as their habits were too entrenched: “It[’s] already my habit… I want to live my life in a better way and healthier. But, *yaa*, it’s only desire, I don’t know if I can do it or not” (male participant, age 21).

This ‘inertia’ regarding those who did not wish to change, or did not believe that they could change, is a recognised challenge of E-E interventions and can be overcome by featuring characters that the audience views as similar to them, or that they would be likely to socialise with [3]. In the case of *RATW*, the primary characters (Rama and Ravana) are stock characters of *wayang kulit* that were selected for their familiarity to Javanese audiences. While Rama and Ravana are deities, they are accompanied in the film by *punakowan* (clown-servants) whose purpose is to ‘ground’ the messages imparted by their lofty masters, making them more attainable. However, based on the participants’ responses, it is possible that an expanded use of such characters (or the incorporation of more relatable village figures) could further improve the efficacy of this intervention.

Positive improvements in participants’ habits since watching *RATW* in general related to washing hands, either before eating (n = 4), after using the lavatory (n = 2), or using soap to do so (n = 1). However, Rama’s other messages (such as that of using the lavatory, keeping food clean and boiling water) were not reflected in the participants’ responses. This was likely due in part to participants’ assertions that they already practiced positive hygiene habits, especially that of covering (n = 8) or washing (n = 4) food, when using the lavatory (n = 5), or washing hands before eating (n = 6), or after using the lavatory (n = 6).

The negative habit that was most frequently identified by participants was either failing to use (n = 2) or inconsistently using (n = 3) soap when washing hands. This did not appear to be due only to a lack of desire to use soap, but also due to a lack of accessibility to it, with one participant commenting that not using soap was less from a lack of desire to do so, but “because there was no soap and I get used to not use it” (female participant, age 16).

For one participant, a lack of public bins appeared to contribute towards their negative habits: “Because when you buy a snack on the street side, you can directly eat while walking, so *yaa*. Sometimes there were no trash can, so *yaa* just throw away” (male participant, age 21). Both of these factors highlight the importance of considering the broader environment beyond the participants’ control [14].

### 3.3. Perceptions of the Music

The majority of participants’ comments regarding the music were predominantly positive (n = 6) or neutral n = 2), with three participants voicing some criticisms. Positive comments that recurred among the participants was that the music was “good”, with no further explanation (n = 5). Additional comments included that they liked the novelty or difference of the music (n = 5), that they enjoyed the combination of modern and traditional music (n = 4), and that the music was better for young people (n = 2). One participant, for instance, commented that the music used in *RATW* was effective because:

Nowadays, teenager must be lazy and bored to watch wayang with gamelan music. They heart would be happy to watch wayang with jazz music that was played yesterday … I think I prefer combination of gamelan and jazz music… *Ya*, the music that was played yesterday is very good *mas* [nickname for older man], because make me feel back to young again.

(Male participant, age 29)

Thus, the reasons why participants enjoyed *RATW* were interlinked, with the modern/traditional fusion enjoyed not only in and of itself, but also in the perceived wider appeal to both the younger and older members of the community. However, the music was not always perceived as a combination of ‘modern’ and ‘traditional’, despite the inclusion of traditional instruments in the music. For instance, one participant commented that “the music was so modern, if it is performed at certain village, no one will watch it—unless using traditional music like Javanese music” (male participant, age 50). Five of the participants commented that *RATW* would be better with traditional music, with four suggesting that this should specifically be gamelan percussion instruments. Another believed that fusion should be avoided, out of a concern that “it will be better if you don’t mix them because the traditional style will be lost” (female participant, age 43). More broadly, one participant highlighted a perceived divide between their village, and larger cities, believing that the music and the narration should be adapted to each province:

I think, we live in Java right? *Ya*, the *wayang* usually use gamelan, even more we live in the village, so, it should be using gamelan and Javanese language. Maybe will be more different when it’s national scale, it will be good using modern way, the modern music and using Bahasa language… We should adapt, like using Bali language when we are in Bali, and so. We are in the village, so we should use native language.

(Male participant, age 29)

### 3.4. Perceptions of the Story

The participants’ preference for adherence to tradition extended to the story elements. Traditionally, *wayang kulit* can run to many hours in length, and four of the participants picked up on this, stating that there weren’t enough scenes. As one participant stated: “It’s only 24 min. Make the story longer, the minimum for one story is one hour” (male participant, age 43). While there is a long tradition of *wayang kulit* being used for all sorts of contemporary purposes in Indonesia, four of the participants proposed that more traditional elements for the story were needed, with two remarking upon the difference in themes: “The story of general *wayang* usually not about health, the possibility is about ancient age… about history, not about health” (male participant, age 49).

The importance of the health messages was reflected in the scene that participants found most exciting—four participants found it to be when Rama, the hero character, fights and defeats the ‘worm’ in the stomach of a boy, although two participants preferred the action scene where Rama fights and defeats Ravana, the traditional villain. In terms of health education, three participants stated that the most exciting point for them was when Rama provides the villagers with health advice. One of these said that the most exciting part of the story was “Rama’s messages—or it can be said that it’s advice for people, because it’s very useful for me and my family and also audiences who watched it.” Despite the mixed response from participants to the music and story elements, the enthusiasm exhibited for the health messages within the drama indicates that such health interventions have promise.

### 3.5. Effect of Age and Gender on Reception and Efficacy of RATW

We investigated both age and gender to identify any themes that would benefit from further investigation, using cross tabulations on frequently recurring codes. Neither gender nor age appeared to have a striking effect on the reception and efficacy of the intervention.

#### 3.5.1. Gender

In most cases, gender appeared to have little impact on the intervention. However, the only participants to state that they preferred receiving health messages through *wayang kulit* (n = 3) were male, preferred over all other methods combined. Conversely, women tended to prefer direct explanations, from those who were professionally trained in health services (such as midwives). This could perhaps be related to stereotypical gender roles, an aspect that arose from some of the interviews. For instance, one participant affirmed that some mothers washed clothes in the river, while another (referring to covering food) stated “I never paid attention whether it’s closed or not, because my mother always manage it” (male participant, age 21). Similarly, when asked about food hygiene, one participant stated that they were “not sure because my wife is the one who cook” (male participant, age 43). Another participant remarked, “I always told to my wife that she has to wash the food first, and wash the plate also to prevent disease and illness” (male participant, age 29). While this participant did indicate that they were taking some level of involvement, here (and in the previous cases) the majority of the responsibility for these areas appears to be placed upon the women. These gendered expectations are worth considering in future E-E interventions.

#### 3.5.2. Age

Age also appeared to have little impact on most aspects of the intervention. However, the only participants who did not remember any of Rama’s main messages (n = 2) were from the older adult category, while all others remembered at least parts of the message. The reason for this was not discernible from the interview data. Participants from the adult category appeared to be the most open to changing their habits after viewing *RATW*, as all from this group expressed the desire to change. The young and older adult participants were equally divided, perhaps reflecting that the young were already practicing better hygiene, while the older adults were more fixed in their ways.

## 4. Discussion

A key moment in *RATW* is when Rama outlines the following three main messages to the townsfolk of the village:

Firstly, you must ensure that poo goes in a place where it is contained, not just out in the open. This is most important. If you do this, the parasites will be trapped and unable to move around in your environment. Secondly, you must wash your hands whenever you go to the toilet. And always make sure your hands are clean before you touch any food you wish to eat. Thirdly, fresh food and water needs to be clean, to make it harder for the worm to return. Make sure you get water that is clean and fresh, sometimes it may need to be boiled first to make sure the invisible germs are gone.

This information, which comes at the end of the film when Rama is addressing the whole community, was considered by the project’s designers to contain the core messages and address the most important hygiene/health promotion goals. It was anticipated that although these messages were delivered as a set of instructions between characters in the narrative as part of the story line rather than as direct hygiene instructions to the audience, they would still carry weight in terms of health education. This was found to be the case, with nine of the eleven participants remembering at least part of the message. The most commonly remembered main messages from *RATW* were the importance of washing hands, keeping the environment faeces free (to prevent getting worms), promoting the awareness of health issues, and maintaining ‘cleanness’ (to avoid worm infection). It is possible that for the two participants who could not recall any of part of the message, the messaging was not sufficiently clear or differentiated [5].

A careful consideration of the culture of the targeted audience of entertainment-based health interventions is crucial to the success of that intervention, however, there is little research on the effectiveness of interventions that incorporate traditional art forms native to non-Western target audiences. This paper helps to address this gap, through investigating the response of villagers to the use of a traditional art form (shadow puppetry) to relay a message designed to combat parasitic infection in central Java and the receptiveness of a village community to this intervention. Our highly experienced *dhalang* (puppeteer) came from central Java, which was critical to the success of the production and this project gives us the opportunity to build a scaled up production building on the advice received from the local community. 

An appropriate model to scale up this project to cover the general Javanese rural population has been provided by the “Magic Glasses” project mentioned above [9]. The cluster-RCT study design used in Hunan, China, allowed for a rigorous quantitative evaluation. In support of a move to scale up the project across Java, the video has been made available in a number of formats, to promote flexibility, and a ‘language free’ format was made so that, whatever the local language, it can be recorded onto the video. Thus, in scale-up terms, it can be used wherever wayang kulit is culturally accepted and popular. More generally, the results reported here can guide the development of future entertainment-based health interventions that aim to use traditional media to improve the health of communities in resource-poor, disadvantaged areas.

The project has a number of limitations, the main one being in terms of scale or size. The study was designed to investigate the acceptability and structure/content of an innovative medium for health education. As such, minimal insight is offered into behavioral change as this would take a far larger project across a longer time span. In addition, the project was designed to test one component of what will be a multi-intervention comprehensive approach to reduction of soil-transmitted helminth infection, which would include construction of ‘improved latrines’ in households, administration of medication (albendizole), engagement of health professionals in support of healthy decision-making, and improved water supply. In terms of data collection, the apparent change of mind demonstrated by a number of participants may have been stimulated by the unnecessary use of probes after the interviewee had already provided their response. This may have led to participants feeling that they should reverse their opinions, or provide the expected or ‘correct’ response.

## 5. Conclusions

This research has been focused on cultural and artistic issues around the legitimacy of using traditional performance for health education purposes, and the cultural meaning of *wayang kulit* for participants. We have noted, and attempt to address, the lack of research on appropriate culturally embedded interventions in countries with a high burden of STHs. Results from our project suggest that there is substantial potential for incorporating traditional media into education-entertainment interventions, although this is not without its challenges. *RATW* was designed to navigate a fine line between featuring familiar elements (such as the incorporation of traditional theatre, story lines, and music), and more modern elements (the incorporated information on STH, and modern instruments). For the participants involved in this small sample, this approach had mixed results, with both positive and critical responses to different aspects of the musical and narrative fusions and no singular consensus on which approach would be best. Significantly, the participants did not identify any specific element of *RATW* that was considered (by the majority) to be unwelcome, incorrect, or objectionable. Those who preferred traditional music or narrative were less likely to exhibit a desire to change, while those open to the modern music fusion all either intended to, or already had, implemented changes as a result of watching *RATW*.

While basing the narrative around the traditional *Ramayana* provided a familiar touchstone for the audience, this did create challenges in conveying the health message. Due to the traditionally fictional nature of the *wayang kulit* stories, certain participants found it difficult to distinguish between what was real, and what was fiction. Additionally, while Rama (as a prince, and an avatar of a god) can be seen as an authority figure likely to inspire compliance with the film’s messages, this character potentially lacks identifiability—a key element in characters designed to inspire behavioral change [5]—and it is possible that the health messaging could be improved through greater prominence of everyday characters in the narrative.

Overall, for scale-up considerations, these findings indicate that the cultural fusion attempted in *RATW* is worth pursuing, as it created a variety of benefits, including increased engagement across different age groups, and the ability of the ‘different’ music to later trigger memories associated with continuing positive behavior. Findings also provide direction for future research, demonstrating the importance of comprehensively understanding both the traditional media to be used, the mechanisms underlying the reception of health messages and the target audience’s motivation to change.

In summary, this research highlights the importance of taking the total health environment of the individual—including, in this context, villagers’ propensity to accept inadequate latrines as being ‘sanitary’ [11]. Overall, the participants indicated general enthusiasm for the incorporation of health messages into their traditional media, although a number of modifications would both improve content and strengthen communication of such health messages. The project shows the promise of adapting such culturally appropriate local art forms for disseminating information on health and social issues.

## Supplementary Materials

The following are available online at http://www.mdpi.com/1660-4601/15/12/2829/s1, File S1: Guided interview questions/prompts, Video S1: ‘Rama and the Worm’.
