# Supplementary material for: Shadow Puppets and Neglected Diseases (2): A Qualitative Evaluation of a Health Promotion Performance in Rural Indonesia
Source: Int J Environ Res Public Health. 2018 Dec 12;15(12):2829. doi: 10.3390/ijerph15122829 (PMC6313519; doi:10.3390/ijerph15122829)
Supplement: Supplementary file 1 [file ijerph-15-02829-s001.pdf]

Appendix: Guided interview questions

1. **What do you think are the main messages from the video 'Rama and the Worm'?**

   (Probes: Any more? Most important message?)

2. **How did you like the use of the Ramayana story to describe the problems in the village?**

   (Probes: Prefer wayang to present traditional Ramayana stories? OK to have stories about modern issues or problems?)

3. **'Rama and the Worm' has music that is different to normal: was this good, or would you prefer more traditional music?**

   (Probes: Can you say anything about what you liked or didn't like about the sound track? Think that the modern music fitted the modern story OK?)

4. **What was the most exciting part of the story?**

   (Probes: What made it exciting? what else was exciting? Did the music add to the excitement?)

5. **Do you think you will do anything different as a result of seeing the video and hearing Rama's advice?**

   (Probes: Any changes in where you defecate? Do you wash your hands more now after going to toilet? Cover your food more now to keep away the flies? Go to get medical treatment if you think you have worms?)

6. **Do you prefer to hear messages about your health from the wayang kulit or if you were given a pamphlet? Or lecture/presentation?**

   (Probes: should health messages only be given by health workers/nurses/doctors? Do you think you learned more from the video than you might have learned from a pamphlet? How do you think you could have improved the video?)

7. **Any other comments about the video?**

   (Probe: anything else? Pause/wait for response.)
